# Astrocytes in the glioblastoma tumor microenvironment

**DOI:** 10.1093/neuonc/noaf191

**Published:** 2026-02-16

**Authors:** Camilo Faust Akl, Brian M Andersen, Francisco J Quintana

**Affiliations:** Ann Romney Center for Neurologic Diseases, Brigham and Women's Hospital, Harvard Medical School, Boston, Massachussetts, USA (C.F.A., B.M.A., F.J.Q.); Ann Romney Center for Neurologic Diseases, Brigham and Women's Hospital, Harvard Medical School, Boston, Massachussetts, USA (C.F.A., B.M.A., F.J.Q.); Department of Neurology, Veterans Affairs Medical Center, Harvard Medical School, Boston, Massachussetts, USA (B.M.A.); Ann Romney Center for Neurologic Diseases, Brigham and Women's Hospital, Harvard Medical School, Boston, Massachussetts, USA (C.F.A., B.M.A., F.J.Q.); The Broad Institute of Harvard and MIT, Cambridge, Massachussetts, USA (FJ.Q.); The Gene Lay Institute of Immunology and Inflammation, Harvard Medical School, Boston, Massachussetts, USA (FJ.Q.)

Therapeutic interventions in glioblastoma (GBM) show little efficacy for multiple reasons, one of which is the highly immunosuppressive nature of the tumor microenvironment (TME). GBM cells interact with neurons and other cells in theTME, such as macrophages, promoting local immunosuppression, tumor invasion, and growth. However, little is known about the relevance of GBM-astrocyte interactions, which exist at the invasive borders of GBM.^1^

Bidirectional functional interactions between astrocytes and other cells in the central nervous system (CNS) control the local immune response, as well as astrocyte heterogeneity.^2,3^ In recent studies, we identified astrocyte subsets that limit the immune response to GBM through their interactions with either T cells or tumor cells. These findings identify novel mechanisms of immunosuppression in GBM, as well as candidate therapeutic targets to remodel the TME and boost protective immunity to GBM.

In our first study,^4^ we used single-cell RNA sequencing to identify in GBM clinical specimens an expanded subset of astrocytes expressing the TNF-related apoptosis-inducing ligand (TRAIL, encoded by *Tnfsf10)*. High TRAIL expression was associated with earlier recurrence and shorter patient survival, prompting further investigations in multiple mouse GBM models.

In mechanistic studies, we established that astrocyte-specific genetic inactivation of *Tnfsf10* boosted the antitumor immune response and extended the survival of glioma-bearing mouse in a T-cell-dependent manner. Further, we found that TRAIL + astrocytes induce T-cell apoptosis, recapitulating previous observations we made in the context of CNS autoimmunity.^5^ In additional studies, we identified the IL-6 family cytokine IL-11 produced by GBM cells as an inducer of TRAIL expression in astrocytes via the activation of STAT3 signaling. Indeed, high IL-11 expression is associated with worse GBM prognosis and has previously been described to potentiate tumor growth by modulating the TME. The inactivation of IL-11R on astrocytes or IL-11 overexpression in GBM cells extended or shortened survival, respectively, by controlling the number of TRAIL + astrocytes in theTME.

Lastly, we therapeutically targeted TRAIL + astrocytes using an oncolytic herpes simplex virus-1 (oHSV) virus designed to infect and lyse GBM cells while also producing aTRAIL-blocking antibody in the TME. TRAIL blockade improved the therapeutic activity of oHSV, enhancing the response of T cells and myeloid cells to GBM. In summary, this study identifies a novel role of astrocytes in the TME, identifying a subset of TRAIL + astrocytes induced by IL-11 that suppresses T-cell responses.

In a second study,^6^ we investigated immunosuppressive astrocyte functions in GBM using a virus-based molecular barcoding technology adapted to detect cell-cell interactions in human tissue (RABID-seq^7^). RABID-seq combines viral interaction tracing with scRNA-seq, enabling the unbiased investigation of cell-cell interactions in the TME of clinical samples at single-cell resolution. Annexin-A1 (ANXA1) expression was highly enriched in scRNA-seq of GBM-associated astrocytes, suggesting a role in the control of the immune response in the TME because ANXA1 is reported to mediate immunosuppressive mechanisms downstream of glucocorticoid signaling. RABID-seq analyses of freshly resected GBM specimens and preclinical models established that ANXA1 + astrocytes mainly interact with GBM cells, in which expression of the ANXA1 receptor formyl peptide receptor (FPR1) is associated with worse clinical outcomes. Indeed, we detected immunosuppressive signaling in ANXA1 + astrocytes that interacted with FPR1 + GBM cells. Conversely, necroptosis signaling in astrocyte-interacting GBM cells was reduced. In follow-up studies, we established that bidirectional astro- cyte-GBM ANXA1-FPR1 communication limits the immune response to the tumor. First, FPR1 activation by ANXA1 in GBM cells inhibits necroptosis, a highly immunogenic form of cell death. Second, reverse ANXA1 signaling in astrocytes suppresses inflammasome activation and inflammatory signaling via NF-kB. The genetic inactivation of ANXA1 in astrocytes or FPR1 in tumors extended survival in multiple GBM models by enhancing the response of tumor-specific T cells and myeloid cells. Thus, astrocyte-GBM ANXA1-FPR1 signaling limits immune responses in the GBM microenvironment and may also promote GBM invasion. In addition, RABID-seq offers a unique opportunity for the study of the TME and the identification of novel targets for therapeutic intervention.

Our efforts also identified challenges related to the study and future therapeutic targeting of GBM-astrocyte interactions.

First, syngeneic animal models allow for the investigation of immune-TME interactions, while being limited in their ability to recapitulate human GBM dynamic cell states, invasiveness, and heterogeneity. Patient-derived models, however, preclude the investigation of immune system interactions with local TME cell types, such as astrocytes. Thus, improved experimental model systems are needed for the study of the GBM TME. Second, GBM tissues collected at surgery for research do not always include invasive edges. When possible, normal-appearing brain specimens from surgeries, when removed due to clinical necessity, ought to be investigated as often as grossly abnormal tissue, due to the need to characterize cell-cell interactions during invasion. Third, shared gene expression between astrocytes and GBM cells often leads to the misclassification of astrocytes as malignant cells in transcriptomic studies. In this regard, improved detection of malignant cells through inference of genetic alterations from transcriptomic data may uncover previously undescribed astrocyte subsets.

Finally, the therapeutic targeting of astrocytes in GBM (and other disorders) is still in its infancy. Such targeting must take into account the challenge posed by the blood- brain barrier. Brain-penetrant drugs and oncolytic viruses are two potential approaches, with the latter being particularly attractive due to their potential to locally deliver combinatorial immunomodulatory payloads (F. Giovannoni, C.A. Strathdee, C. Faust Akl, unpublished manuscript).^8^ For these axes to be safely targeted, further preclinical work is required investigating the impact of therapeutic strategies on the interaction between astrocytes and various other cell types, such as neurons, or tumor-associated microglia- derived or monocyte-derived macrophages.^9^
